# Motor development of infants exposed to maternal human immunodeficiency virus (HIV) but not infected

**DOI:** 10.1186/1755-7682-6-45

**Published:** 2013-10-31

**Authors:** Dafne Herrero, Paulo Rogério Gallo, Mahmi Fujimori, Carlos Bandeira de Mello Monteiro, Vitor E Valenti, Carlos Mendes Tavares, Sophia Motta Gallo, Cícero Cruz Macedo, Adriana G Oliveira, Luiz Carlos de Abreu

**Affiliations:** 1 Departamento de Saúde Materno-Infantil da Faculdade de saúde, Pública da Universidade de São Paulo, São Paulo, Brasil; 2 Laboratório de Delineamento e Escrita Científica. Departamento de Ciências Básicas, Faculdade de Medicina do ABC, Av. Príncipe de Gales, 821, 09060-650, Santo André, SP, Brasil; 3 Departamento de Fonoaudiologia, Faculdade de Filosofia e Ciências, UNESP, Av. Hygino Muzzi Filho, 737, 17525-900, Marília, SP, Brasil; 4 Universidade da Integração Internacional da Lusofonia Afro-Brasileira, Instituto de Ciências Sociais Aplicadas, Campus da Liberdade Centro, 62790000, Redenção, CE, Brasil

**Keywords:** Motor development, Assessment, HIV/AIDS, Early intervention, Physiotherapy

## Abstract

**Background:**

To assess the motor development of infants exposed to maternal human immunodeficiency virus (HIV).

**Methods:**

Thirty infants were assessed in the period from November 2009 to March 2010 at the AIDS Reference and Training Centre, in São Paulo, Brazil. The assessment instrument used in the research was the Alberta Infant Motor Scale (AIMS). All 30 infants used the antiretroviral drug properly for 42 consecutive days, in accordance with the protocol of the World Health Organization.

**Results:**

Out of the total number of infants, 27 (90%) had proper motor performance and 3 (10%) presented motor delay, according to the AIMS.

**Discussion:**

This study demonstrated that only 10% of the assessed group had developmental delay and no relation with environmental variables was detected, such as maternal level of education, social and economic issues, maternal practices, attendance at the day care center, and drug use during pregnancy. It is important to emphasize the necessity of studies with a larger number of participants.

## Background

Motor development has been discussed and more intensively investigated recently [1-3]. Theories try to explain whether the main influence on its progress is the biological maturation or the environmental experience [4], taking into account the importance of the initial bond of the infant with the reference carer [5], and the factors that may cause cultural and clinical interference [6-9].

As to the group of the main genetic and environmental issues that interfere in the child development, it is known that the causes of morbidity related to pregnancy and labor appear more intensively in the first week [10]. During the neonatal period, however, the environmental causes start to appear more frequently in the statistics, expressed mainly through infections [11], which include issues related to social and economic issues, maternal educational level, maternal practices, attendance at the day care center and use of drugs. Neonatal anoxia, low birth weight, neonatal tetanus, and intrauterine infections [10], such as AIDS, are among the main causes of morbidity and mortality of infants.

In developing countries, mainly Latin America countries, HIV infections spread more intensively among women, which consequently increased the vertical transmission rate, that is, the percentage of infections from mother to child [12,13]. In Brazil, more specifically in 1985, the first case of a child with HIV was reported by the Ministry of Health [14,15]. Currently, 30,000 new cases of AIDS appear every year in Brazil and vertical transmission accounts for 80%-90% of the cases of HIV in children [16,17].

There are more vulnerable groups in the population. In order to identify such groups, social, behavioral, and political and institutional aspects are taken into account. These are analyzed together with individual aspects and specific conditions (Matida, 2010) [18]. The transmission rate in developing countries remains increased due to factors such as difficulty of diagnosis during pregnancy, failure to follow the antiretroviral therapy or post-exposure prophylaxis, and failure of total replacement of the breastmilk with infant formula [19,20]. It should be noted that in 1999, 39% of the infants of a mother with confirmed diagnosis were still breastfed and Silva et al, [21] stated that in a study conducted with two groups of infected and non-infected infants, breastfeeding appears as an additional risk of postnatal transmission. Ivers et al. suggest in their study concluded that breast milk substitution was safe, acceptable and feasible for HIV-infected women choosing this option [22].

This vertical or mother-to-child transmission may occur during the intrauterine life, labor, postpartum, mainly during breastfeeding [21]. Statistics show that approximately 65% of the cases occur during labor, 35% occur during the intrauterine period, mainly in the last weeks of pregnancy, and breastfeeding represents an additional risk of transmission of 7% to 22% [23]. Yoshimoto, (2005) [16] also says that the transmission occurs mainly during labor and emphasizes that 70%-90% of the cases are characterized by the late appearance of symptoms in children and adolescents.

Among the *infected* infants and children there may be manifestations such as weight-height deficit (clinical finding in 69.5% of the cases before one year of age), anemia, hypochromia (paleness due to deficiency of hemoglobin), macrocytosis (increase of the size of the red blood cells), atypical lymphocytes, lymphopenia (reduction in the number of lymphocytes), and mononucleosis (increase of the number of monocytes) (Smith et al, 2000) [24]. However, the action of the medication on the development and growth of *exposed* children is still controversial and more research is needed in order to determine what the real risks and benefits are. Therefore, we aimed to verify the motor characteristics of non-infected children born from mothers with medical diagnosis of AIDS.

## Methods

### Study population

The research was carried out after approval by the Independent Ethics Committee of the AIDS Reference and Training Centre and by the Ethics Committee of the Public Health College of USP, in accordance with the rules governing research on human beings of Resolution No. 196/96 and No. 251/97 of the National Health Council.

The aims of the research as well as the procedures were previously explained to all parents or legal representatives. The research was conducted only after the consent forms were obtained from those responsible.

It was a cross-sectional study conducted in an outpatient clinic specialized in assisting patients with HIV/AIDS, from November 2009 to March 2010.

### Infant profile

We investigated 30 full term newborns aged between 20 days and 18 months, out of which 19 were male and 11 were female. The infants were exposed to ARTs in utero and were given prophylactic AZT after birth. The primary exposure was ART prophylaxis. Most of them, 25, were born by cesarean section and 5 were born by normal delivery (the latter being contraindicated in cases of infected mothers). At the moment of assessment, the viral load of the assessed infants was undetectable. They were using or had already made use of the antiretroviral for 42 days after birth (AZT, oral route). The medication received by mothers during pregnancy was the combination of three of the following antiretroviral drugs: tenofovir, neviparine, kaletra, biovir, ritonavir, atazanavir, and AZT, depending on each patient and the acceptance of the combination of such drugs. Mothers who had already been diagnosed with AIDS before pregnancy (10) were already using ARVs. Five out of the remaining (20) used the medication from the first quarter on, thirteen from the second quarter on, and two of them did not use it.

### Assessment tools

Initially, a structured questionnaire was used. It contained important information, such as gender, age, adhesion to the treatment with antiretroviral, number of people living in the same house, place where the infant remains at home, and use of drugs.

The Alberta Infant Motor Scale, an observational scale made by Piper and Darrah (1994) [25] to assess the motor development of infants from birth until 18 months was used to verify the motor development. Some of the objectives of the scale are: (1) identify restrictions of the neuromotor development of infants; (2) inform parents about the motor activities that the baby performs, the activities that the infant does not perform, and the activities that are being developed; (3) analyze the motor development in a certain period of time or before and after hospitalization; (4) measure the very small changes in the motor development which cannot be identified through more traditional methods; (5) act as a research instrument in order to identify the effectiveness in stimulation programs for infants with motor disorders.

The score may be with percentile <5, indicating delayed motor development; between 5 and 10, indicating potential delay and the need for monitoring, and between 10 and 90 percentile, indicating proper development.

After the questionnaire and tests were applied, data were verified through descriptive analysis.

## Results

The characteristics of patients and the results achieved are shown in Table 1, where we observe that out of the 30 infants assessed, only 3 (10%) had a score below the 10th percentile and the remaining 27 (90%) had the percentile expected for the age.

**Table 1:** Data obtained from the structured questionnaire and assessment through Alberta Scale

	**Maternal educational level**	*A to T	**PSH	***PIH	**Day care center**	**Use of drugs**	**Result AIMS**
1	finished college	Y	5	floor	Y	N	Adequate
2	finished college	Y	3	cradle	N	N	Adequate
3	finished high school	Y	3	floor	N	N	Adequate
4	finished high school	Y	9	floor	Y	N	Adequate
5	finished high school	Y	4	floor	Y	N	Delay
6	finished high school	Y	3	floor	N	N	Adequate
7	did not finish high school	Y	4	cradle	N	N	Adequate
8	finished high school	Y	4	stroller	N	N	Adequate
9	unfinished college degree	Y	8	stroller	N	N	Adequate
10	unfinished college degree	Y	3	stroller	N	N	Adequate
11	unfinished high school	Y	4	floor	N	N	Adequate
12	unfinished high school	Y	7	floor	N	N	Adequate
13	finished college	Y	6	floor	N	N	Delay
14	finished high school	Y	3	floor	N	N	Adequate
15	finished high school	Y	6	floor	N	N	Adequate
16	finished high school	Y	4	floor	N	N	Adequate
17	finished college	Y	5	stroller	N	N	Adequate
18	finished high school	N	5	floor	N	Y	Adequate
19	finished high school	Y	3	floor	Y	N	Adequate
20	unfinished high school	Y	4	floor	Y	N	Adequate
21	finished high school	Y	6	bed	Y	Y	Adequate
22	finished primary school	Y	6	bed	Y	N	Adequate
23	unfinished primary school	Y	6	lap	Y	N	Delay
24	unfinished primary school	Y	6	floor	Y	N	Adequate
25	did not study	N	3	stroller	N	Y	Adequate
26	finished high school	Y	2	stroller	N	N	Adequate
27	unfinished college degree	Y	3	stroller	N	N	Adequate
28	finished high school	Y	5	floor	N	N	Adequate
29	finished primary school	Y	12	lap	N	Y	Adequate
30	unfinished high school	Y	4	bed	N	N	Adequate

Y = yes, N = no, *A to T = adhesion to treatment, **PSH = people living in the same house as the infant, ***PIH = place where the infant remains at home.

Table 1 describes that adherence to treatment was verified by the data as an undetectable viral load and antiretroviral that was used in 100% of cases. As for the data received by the group of stimuli, the place of residence in the house was investigated the location where the infant stays (‘spends most of their time’) greatly determines whether it favors the reception of sensory information and diversity of motor experience), day care attendance and the number of people in the house (besides detecting drug users among locations).

## Discussion

The population comprised of infected mothers and infants exposed to HIV during pregnancy and breastfeeding brings, in specific or global terms, certain demands that involve environmental and biological factors related to child development, which are considered harmful or facilitators of such a process [26].

Ribeiro and Beltrane (2010) [27] explain that biological risk factors can interfere in neuromotor development, especially if there is a history of risk from birth up to 18 months. Pretti et al., (2010) [28], however, state that the environmental risk factors are the main harmful effects for motor development.

The mother-child relationship is considered a determining factor for full development of the child and family involvement in the care dedicated to the infant. Paiva et al., (2010) [29] show us that the non-verbal communication present in the situation of the mother with HIV and her child causes attachment to the infant and increases the likelihood of identifying abnormalities in their development. The uncertainty of waiting for the serology results and the overprotection of their children are mentioned by Galvão et al., (2010) [30] as some of the dilemmas and conflicts present in the life of the infected mother.

Fernandes (2010) [19] demonstrates that, in Brazil, there was not a reduction of the mother-to-child HIV transmission rate in the period from 2004 to 2007 and characterized the following as determining obstacles of such non-reduction: the low coverage for anti-HIV testing during prenatal care, preventing treatment or efficient maternal prophylaxis; and the incorrect use of rapid testing in the admission for delivery. Although there is prenatal exposure to a drug, it is not possible to determine the immediate results in a child, but such exposure can be a marker for a number of variables that can have impact on development. Proper intervention strategies require investigations to determine which factors that expose children to the risk and protect the ideal development [31].

Ribeiro and Beltrane, (2010) [27] mention that children with biological risk history may have some impairment regarding development aspects. According to Jelsma et al., (2011) [32] children with HIV were significantly delayed when compared to their HIV-negative counterparts. Attention, motor function, and executive function were particularly compromised among individuals with HIV [33].

In the data shown in this study, it was identified that only 3 (10%) out of the 30 infants presented delay in the assessment instrument used and the influence of the use of antiretroviral was not detected. That is, the prophylactic use of antiretrovirals during the periods indicated as being most suitable is one of the best strategies for preventing AIDS in childhood [34,35] and probably it does not imply delays in motor development. Yoshimoto, 2005 suggests that the prophylaxis with AZT in all recommended periods and long term follow-up of infants born to HIV positive mothers constitutes one of the best strategies for preventing AIDS in childhood.

The use of drugs associated to the stimulus received at home, at the day care center, or places attended by the infant helps in preventing delay in motor and full development. The environmental factors were also assessed and are discussed below

a) maternal educational level: according to Haidar et al., (2001) [36], the maternal educational level can be considered an obstetric marker of risk for the mother and the newborn. Knowing the maternal perceptions about AIDS can provide health professionals with more understanding about their behavior. The group also demonstrates that the educational level is related to nutritional matters. Infant formula is offered to replace breastmilk in order to prevent vertical transmission [30]. However, information and family participation are fundamental. Machado, (2010) [34] mentions that it is important to investigate the vulnerability to possible infant food deficit and likely issues such as proper information to mothers, understanding the guidance provided by health professionals, care when preparing the formula, access in the quantity indicated by the Ministry of Health, and availability at the points of delivery are factors that contribute to make proper nutrition difficult. In this study, the maternal educational level was investigated. The three infants with developmental delay had mothers with different educational levels, thus, did not show any relationship in this study.

b) social and economic issues: unemployment, poverty, difficult access to health services and education, and the number of people living in the house are factors that may contribute to characterize economic difficulties. Barcellos et al., (2009) [37] mention that urban poverty represents a strong conditioning factor for vertical transmission of HIV. However, the action of health surveillance services in association with the basic attention can overcome this tendency. In Brazil, it is necessary to have prevention strategies and health promotion, taking into account the social context of the families living with HIV/AIDS [34] During the investigations of these factors, the possibility of any relationship between social and economic issues and the three infants with developmental delay (Table 1) was also not detected.

c) maternal practices and attendance at the day care center: maternal practices can influence the progress of infant development, for example, the position in which the infant stays during the day [3] and these practices change according to the local culture [38-41]. North-American infants stay more in prone, with differences found in other locations, such as British infants in supine, African infants are encouraged to sit and walk early, Mexican infants spend much time on the lap and Brazilian infants remain more in supine than in prone [3]. Mei, (1994) [41] demonstrates that behavioral manifestations observed in infants in the North of China lead to a restriction of child mobility causing interference in motor development. The act of attending a day care center should also be taken into account in the infant development. Identifying whether the infant goes to a day care center provides information on how stimulated he/she is. According to Rezende et al., (2010) [42], infants improve their motor development after they start to attend the day care center and Amorim et al., (2010) [43] show us that the non-participation in a day care center associated to the decrease of the time spent with the mother are linked to the delay in motor development. By analyzing the results of this study, questions related to the most frequent position of the infant at home and whether the infant spends certain time at the day care center enabled different answers and it was not possible to detect any relationship.

d) use of drugs: in the studies conducted by Negrini, (2004) [26] and Eyler et al., (1999) [31] it was observed that there may impairment in the development of infants whose mothers use drugs during pregnancy. They add that such fact can be an opening for the appearance of more factors of risk to the development. The use of drugs by at least one family member of the assessed infants appeared in seven out of the 30 assessments. However, this group mostly presented with appropriate motor development, according to the evaluation criteria to the Alberta Scale. Additionally in this context, longitudinal infants are necessary by the number of factors that can affect the course of their motor skills.

Although environmental influence is crucial to the development of the infant, it is important to emphasize that some of these factors were verified in this study, but the number of individuals with delay did not enable any correlation test. In the descriptive analysis, there was not any possible inferred relationship between the assessed environmental factors, maternal educational level, social and economic issues, maternal practices, attendance at day care center, and the use of drugs with the delay in development.

Our study presents some points that are important to be raised. The number of infants studied (30) is too small to extract consistent conclusions. We suggest future investigations. The educational and social background of the mothers is too heterogeneous, while the longitudinal aspect for this group was only until the age of two.

## Conclusion

It was not possible to verify the influence in the development delay of the assessed group based on the environmental variables assessed (maternal level of education, social and economic issues, maternal practices, attendance at the day care center, and use of drugs).

## Competing interests

The authors declare that they have no competing interests.

## Authors’ contributions

DH, PRG, MF, CBMM, VEV, CMT, SMG, CCM, AGO and LCA participated in the acquisition of data and revision of the manuscript. VEV, DH, LCA, CMT, AGO, SMG and PRG authors conceived of the study, determined the design, performed the statistical analysis, interpreted the data and drafted the manuscript. All authors read and gave final approval for the version submitted for publication. All authors read and approved the final manuscript.
